# Ternary Copper(II) Complexes With Indomethacin, a Potent Non-Steroidal Antiinflammatory Drug. Crystal Structure of Bis (Dimethylformamide)-Tetrakis[1-(4-Chlorobenzoyl)-5-Methoxy-2-Methyl-1-*H*-Indole-3-Acetato]Dicopper(II). Antiinflammatory Properties and Prevention of Gastrointestinal Side Effects by Nanocapsules

**DOI:** 10.1155/MBD.1998.337

**Published:** 1998

**Authors:** Fadila Guessous, Jean-Claude Daran, Bernard Viossat, Georges Morgant, Xavier Labouze, Anne Laure Leroy, Monique Roch-Arveiller, Nguyen-Huy Dung

**Affiliations:** 1 CNRS URA 1534 Laboratoire de Pharmacologie Hôspital Cochin Paris France; 2 Laboratoire de Chimie de Coordination UPR-CNRS 8241 Campus 205 Toulouse France; 3 Laboratoire de Chimie Générale Faculté de Médecine et de Pharmacie Poitiers France; 4 Laboratoire de Cristallochimie Bioinorganique Faculté de Pharmacie Paris XI Châtenay-Malabry France; 5 Laboratoire de Biochimie Hôspital Armand Trousseau Paris AP-HP France; 6 Laboratoire de Pharmacie Galénique Université Claude Bernard Lyon France; 7 Laboratoire de Biochimie Faculté des Sciences El Jadida Morocco; 8 Laboratoire de Cristallochimie Bioinorganique Faculté de Pharmacie 5 rue J-B Clément Châtenay-Malabry Cedex F-92296 France

## Abstract

Two ternary copper(ll) complexes of indomethacin [1-(4-chlorobenzoyl)-5-methoxy-2- methyl-1-H-indole-3-acetic acid] called hereafter lndo, were prepared and characterized by single crystal X-ray diffraction. The first complex Cu_2_(Indo)_4_(DMF)_2_ I crystallizes in space group P-1 (a = 10.829(2), b = 13.379(2), c = 16.491(3) Å; α = 105.58(2), β = 101.06(2), γ = 106.96(2)°; V= 2104.6(6) Å^3^, Z= 1). The title molecule is a centrosymmetric binuclear complex, with Cu atoms bridged by the carboxylate moieties of four indomethacinate ligands. The four nearest O atoms around each Cu atom form a square planar arrangement with the square pyramidal coordination completed by the O atom of N,N′-dimethylformamide. Daily administration for seven days of 1 mg/kg of indomethacin, **I** and **I** encapsulated into liposomes induces a weak inflammation of rat gastrointestinal tract. **I** was less inflammatory than indomethacin but the better protection was brought by encapsulation of the compound. This might be of interest in sustained therapies of chronic inflammatory diseases.


### TERNARY COPPER(II) COMPLEXES WITH INDOMETHACIN, A POTENT NON-STEROIDAL ANTIINFLAMMATORY DRUG. CRYSTAL STRUCTURE OF BIS (DIMETHYLFORMAMIDE)-TETRAKIS[1-(4-CHLOROBENZOYL)-5-METHOXY-2-METHYL-1-*H*-INDOLE-3-ACETATO]DICOPPER(II). ANTIINFLAMMATORY PROPERTIES AND PREVENTION OF GASTROINTESTINAL SIDE EFFECTS BY NANOCAPSULES

Fadila Guessous[1,7], Jean-Claude Daran[2], Bernard Viossat[3], Georges Morgant[4,5], Xavier Labouze[4], Anne Laure Leroy[6], Monique Roch-Arveiller[1] and Nguyen-Huy Dung[4]*.

[1] CNRS URA 1534, Laboratoire de Pharmacologie, Hôpital Cochin, Paris, France

[2] Laboratoire de Chimie de Coordination UPR-CNRS 8241, Campus 205, Toulouse, France

[3] Laboratoire de Chimie Générale, Faculté de Médecine et de Pharmacie, Poitiers, France

[4] Laboratoire de Cristallochimie Bioinorganique, Faculté de Pharmacie Paris XI, Châtenay-Malabry, France

[5] Laboratoire de Biochimie, Hôpital Armand Trousseau, AP-HP, Paris, France

[6] Laboratoire de Pharmacie Galénique, Université Claude Bernard, Lyon, France

[7] Laboratoire de Biochimie, Faculté des Sciences, El Jadida, Maroc

* Correspondence and reprint requests: E-mail: dung.nguyen@cep.u-psud.fr.
Laboratoire de Cristallochimie Bioinorganique, Faculté de Pharmacie,
5 rue J-B Clément. F-92296 Châtenay-Malabry Cedex. France.

**Abstract**

Two ternary copper(II) complexes of indomethacin [1-(4-chlorobenzoyl)-5-methoxy-2-methyl-1-H-indole-3-acetic acid] called hereafter Indo, were prepared and characterized by single crystal X-ray diffraction. The first complex $Cu_2(Indo)_4(DMF)_2$ **I** crystallizes in space group P-1 (a = 10.829(2), b = 13.379(2), c = 16.491(3) Å; $\alpha$ = 105.58(2), $\beta$ = 101.06(2), $\gamma$ = 106.96(2)°; V= 2104.6(6) $Å^3$, Z= 1). The title molecule is a centrosymmetric binuclear complex, with Cu atoms bridged by the carboxylate moieties of four indomethacinate ligands. The four nearest O atoms around each Cu atom form a square planar arrangement with the square pyramidal coordination completed by the O atom of N,N'-dimethylformamide. Daily administration for seven days of 1 mg/kg of indomethacin, **I** and **I** encapsulated into liposomes induces a weak inflammation of rat gastro-intestinal tract. **I** was less inflammatory than indomethacin but the better protection was brought by encapsulation of the compound. This might be of interest in sustained therapies of chronic inflammatory diseases.

### 1. Introduction

Alzheimer's disease (AD), the third most costly disease after cancer and heart diseases [1], is a neurodegenerative disorder, the primary or neuropathogenic mechanism of which is unknown. This disease is characterized by abnormal accumulation of amyloid peptide ($\beta$ A4) from its precursor amyloid precursor protein. Mutations in the gene encoding amyloid precursor on chromosome 21 have been found to segregate with AD [2]. The inflammatory hypothesis of Alzheimer' s disease is based on the observation that individuals susceptible to the disease and who are undergoing treatment for a known inflammatory injury will be inadvertently treating their latent AD and will therefore have a reduced risk of developing a clinical case [3-4]. It was hypothesized that antiinflammatory drugs such as NSAIDs may slow the onset or progression of AD by reducing inflammation in brain due to the deposition of amyloid protein and consequent damage to brain cells. Activated microglia are found to be present in AD [5] and associated with the generation of components of the classical complement pathway, demonstrating the onset of an inflammatory process. Moreover, some of these components stimulate the respiratory burst system of the microglia, resulting in the production of toxic reactive oxygen species such as superoxide anion, hydrogen peroxide and hydroxyl radical [5]. Current clues to possible treatment include the potential use of antioxidants, oestrogens, antiinflammatory drugs or compounds altering amyloid metabolism. Strategy in addressing the stimulation of the immune system has been effective in reducing AD progression in clinical trials related to the cyclooxygenase inhibitor indomethacin [6], which rapidly and efficiently penetrates the blood-brain barrier. While aspirin [7], ibuprofen [8] and indomethacin [9] are at best only very weak free radical scavengers *in vitro* systems, their copper complexes are very efficient free radical scavengers [10]; these drugs are thus expected to circumvent the toxicity of reactive oxygen species generated in the activated microglia.

Even if NSAIDs prove to confer a protective benefit against inflammatory brain disorders, their use is often associated with a broad spectrum of untoward reactions, particularly in the gastrointestinal tract which may constitute a limiting factor for their continuous administration [11]. Copper(II) complexes of NSAIDs may constitute an alternative way. These complexes are extensively studied since Sorenson demonstrated that they are more active than their parent drugs and exhibit antiulcer activity [12,13]. Further, a recent investigation of copper(II) complex equilibria (under physiological conditions) with salicylic or acetylsalicylic acids has shown that their anions may favour the gastrointestinal absorption of copper(II) [14]. In previous work, Sorenson described the synthesis of many copper(II)-NSAIDs complexes and among them, Cu-indomethacin complex [12,13].

In 1980, Weser et al. reported the crystal structures of $Cu_2(indomethacin)_4.L_2$ (L=$H_2O$, DMSO), the latter complex of which was also solvated by DMSO molecules and with an R(F) factor = 0.13 in the final refinement [15]. Thus, a more acurate refinement seemed desirable. This paper presents the synthesis of new ternary complexes $Cu_2(Indo)_4(DMF)_2$ **I** and $[Cu_2(Indo)_4(DMF)_2].2DMF$ **II** and the molecular and crystal structure of **I**.

Moreover, we have previously demonstrated intestinal damage induced by a high dose of indomethacin [16]. In this study, we investigated the effect produced on rat intestine by daily administration of $Cu_2(Indo)_4(DMF)_2$, relative to indomethacin at the commonly prescribed dose of 1 mg/kg. It has been speculated that endogenous nitric oxide (NO) mediates inflammatory processes [17-19]. Nitrite production by intestinal tissues from treated animals has thus been measured in parallel to nitric oxide synthase (NOS) activities. Intensity of the inflammatory reaction was evaluated by measurement of myeloperoxidase activity in the same tissues.

### 2. Results

### 2.1 Description of the structure.

The bond distances and angles in copper coordination spheres are listed in table 1. The molecular conformation of $Cu_2(Indo)_4(DMF)_2$ **I** is illustrated in Figure 1. As the DMF ligand is disordered, only one of its two components is shown.

*Copper environment*

The complex $Cu_2(Indo)_4(DMF)_2$ **I** is dimeric and exhibits a symmetry center : two $Cu^{II}$ atoms are surrounded by two crystallographically independent indomethacinate groups, one DMF ligand, on one side, and by their centrosymmetric counterparts, on the other side. The DMF ligands are coordinated to Cu atoms through the O atom.

The indomethacinate groups are bidentate and act as syn-syn bridging ligands between the isolated pairs of copper atoms separated by $Cu...Cu^i$ : 2.629(2)Å (symmetry code i : -x, 1 - y, 1 - z) in compound **I**. These distances do not differ significantly from the 2.632(6) Å value observed for $Cu_2(Indo)_4.L_2$ (L = $H_2O$ or DMSO) which might be solvated by DMSO molecules **III** [15]. However, these distances are longer than the bond length 2.55 Å in metallic copper [20]. Nevertheless, they lie within the range 2.610 - 2.688(1) Å found in dimeric copper (II) benzoates and are also close to those found in copper-NSAIDs complexes : 2.6265(8) Å in $Cu_2(diclofenac)_4(DMF)_2$, and 2.629(1) Å in $Cu_2(naproxen)_4(DMSO)_2$ [21-23]. The four nearest O atoms around each Cu atom form a square planar arrangement with the copper to carboxylato bond distances in the range from 1.956(7) to 1.967(7) Å in **I**. The square pyramidal environment is completed by the O atom from N,N'-dimethylformamide, at a distance of 2.154(6) Å in **I**. Similar Cu - O(DMSO) or Cu - O(DMF) apical bond lengths are observed for previously reported Cu-NSAID complexes : in the range from 2.123(5) to 2.16(2) Å [15, 22-23]. The Cu atom in **I** lies 0.202(3) Å out of the O atoms least-squares plane towards the DMF oxygen atom. It is well known that the axial ligand plays a role in determining both the Cu - Cu and Cu - O basal plane distances. The O(100) - Cu - $Cu^i$ angle is 177.2(2) and the dihedral angle between the plane through Cu - O(1) - C(1) - O(2) - $Cu^i$ and Cu - O(21) - C(21) - O(22)

- $Cu^i$ is 89.9(2) in **I**. In conclusion, the crystal structures of **I** is essentially the same as in **III** except for the DMF or DMSO accomodation. However, compare to the molecular structure of the indomethacin free ligand [24], there is a conformation change in the second indomethacinate ligand as shown by the O(3) - C(13) - N(1) - C(10) and O(23)-C(33)-N(21)-C(30) torsion values in compound **I**.

| Torsion angles (°) | $[Cu_2(Indo)_4(DMF)_2]$ | Indomethacin free ligand |
|---|---|---|
| O(3)-C(13)-N(1)-C(10) | 24.8 | -25.5 |
| O(23)-C(33)-N(21)-C(30) | 149.0 | |

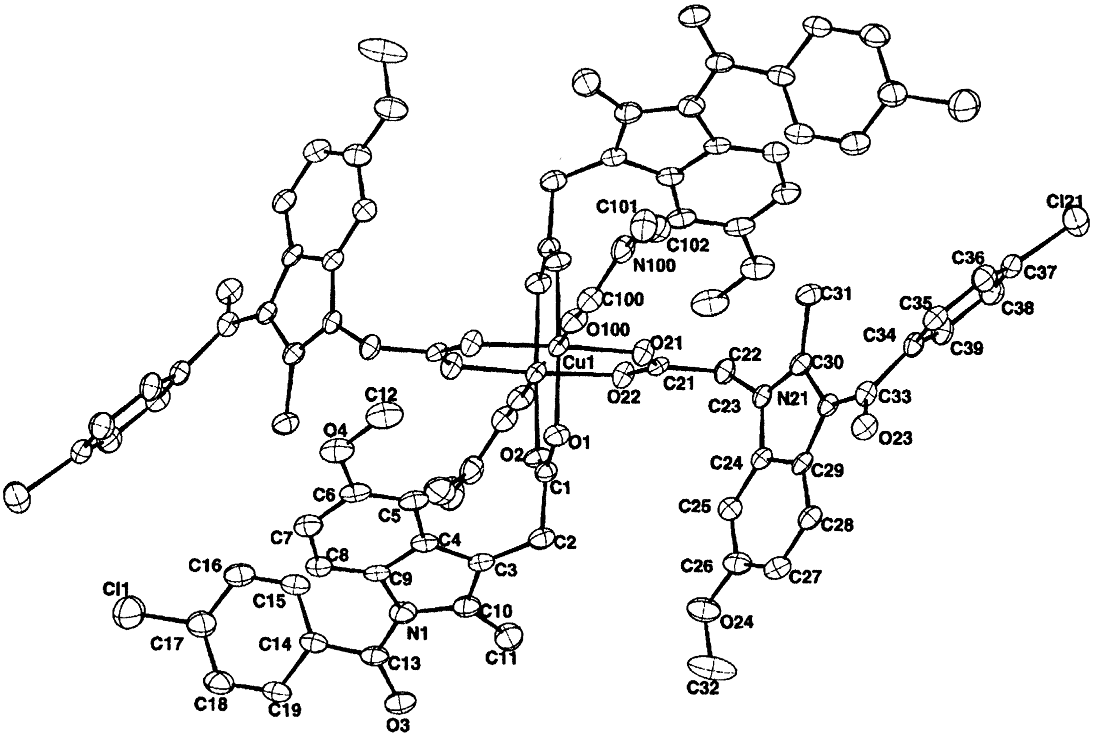

Figure 1 : Perspective view of $Cu_2(Indo)_4(DMF)_2$ generated by CAMERON using 20% probability ellipsoïds for the non-hydrogen atoms. The crystallographic labelling is shown. Hydrogen atoms are omitted for clarity.

### 2.2. Biological properties

### 2.2.1. Intestinal damage score.

Daily administration for seven days of 1 mg/kg of indomethacin and $Cu_2(Indo)_4(DMF)_2$ compound **I** encapsulated or not, did not induce gastric or intestinal damages.

### 2.2.2. Myeloperoxidase activity in intestine tissue.

An inflammatory process has been demonstrated by a significant enhancement of MPO activity in intestinal tissue after administration of NSAIDs (Figure 2). This enzyme released by inflammation cells and particularly polymorphonuclear cells is generally considered as a good marker of inflammatory process and classically measured to appreciate such a phenomenon. Since myeloperoxidase-mimetic activity has been reported with some copper complexes [12-25], similar enhancement of tissue myeloperoxidase content after treatment with indomethacin and copper indomethacinate complex might be partly due to the proper effect of the complex more than to a proinflammatory effect of this compound. Conversely, encapsulation of copper indomethacinate counteracted this increase.

### 2.2.3. iNOS activity and nitrite production in intestine tissue .

iNOS activity and nitrite production were significantly enhanced in intestine tissue after administration of indomethacin but $Cu_2(Indo)_4(DMF)_2$ did not induce as much inflammation (table 2), with a lesser extent when $Cu_2(Indo)_4(DMF)_2$ was used. No significant effect was observed when this compound was encapsulated.

Table 1. Coordination sphere in $Cu_2(Indo)_4(DMF)_2$ **I**

| | Distances [Å] | | Angles [°] |
|---|---|---|---|
| Cu(1)-O(22)$^i$ | 1.956(7) | O(22)$^i$-Cu(1)-O(2)$^i$ | 89.5(3) |
| Cu(1)-O(2)$^i$ | 1.961(7) | O(22)$^i$-Cu(1)-O(21) | 168.3(2) |
| Cu(1)-O(21) | 1.963(6) | O(2)$^i$-Cu(1)-O(21) | 89.5(3) |
| Cu(1)-O(1) | 1.967(7) | O(22)$^i$-Cu(1)-O(1) | 89.1(3) |
| Cu(1)-O(100) | 2.154(6) | O(2)$^i$-Cu(1)-O(1) | 168.1(3) |
| Cu(1)-Cu(1)$^i$ | 2.629(2) | O(21)-Cu(1)-O(1) | 89.5(3) |
| | | O(22)$^i$-Cu(1)-O(100) | 94.0(3) |
| | | O(2)$^i$-Cu(1)-O(100) | 94.7(3) |
| | | O(21)-Cu(1)-O(100) | 97.8(3) |
| | | O(1)-Cu(1)-O(100) | 97.3(3) |
| | | O(22)$^i$-Cu(1)-Cu(1)$^i$ | 83.9(2) |
| | | O(2)$^i$-Cu(1)-Cu(1)$^i$ | 83.6(2) |
| | | O(21)-Cu(1)-Cu(1)$^i$ | 84.4(2) |
| | | O(1)-Cu(1)-Cu(1)$^i$ | 84.5(2) |
| | | O(100)-Cu(1)-Cu(1)$^i$ | 177.2(2) |
| | | C(1)-O(1)-Cu(1) | 123.2(6) |
| | | C(1)-O(2)-Cu(1)$^i$ | 123.6(6) |
| | | C(21)-O(21)-Cu(1) | 122.3(6) |
| | | C(21)-O(22)-Cu(1)$^i$ | 123.5(5) |
| | | C(200)-O(100)-Cu(1) | 111(1) |

Symmetry transformations used to generate equivalent atoms: i : -x,-y+1,-z+1

**2.2.4. Antiinflammatory activity of encapsulated copper indomethacinate.**

Antiinflammatory property of copper indomethacinate was verified by the reduction of carrageenan-induced paw edema in treated animals in comparison with solvent (RPMI + 1% DMF)-treated rats (Figure 3).

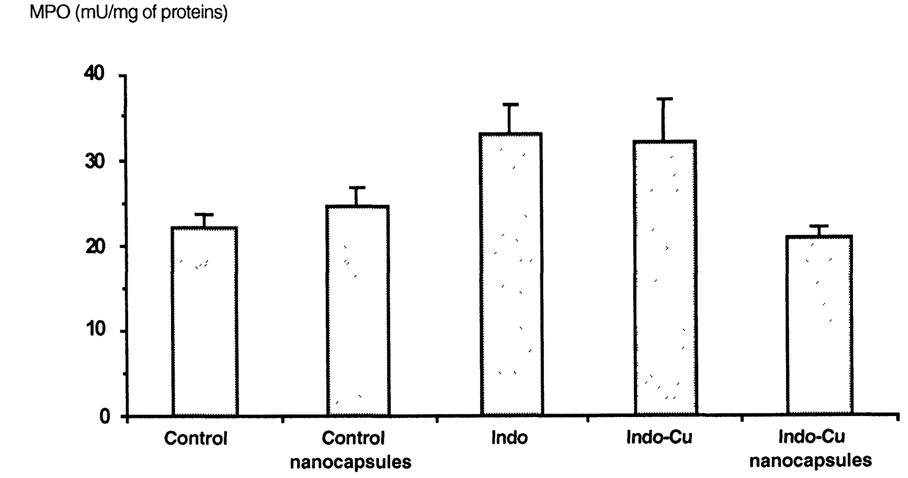

Figure 2 : Myeloperoxydase production in intestine tissue from animals treated by RPMI medium (C), saline containing empty nanocapsules (C nano), indomethacin (indo), $Cu_2(Indo)_4(DMF)_2$ (indo Cu) or encapsulated $Cu_2(Indo)_4(DMF)_2$ (indo-Cu nano).

| | iNOS | Nitrite |
|---|---|---|
| **Control** | 0.1 ± 0.1 | 0.12 ±0.06 |
| **Control nanocapsules** | 0.09 ± 0.09 | 0.09 ± 0.05 |
| **Indomethacin** | 1.0 ± 0.3 | 0.31 ± 0.05 |
| **$[Cu_2(Indo)_4(DMF)_2]$** | 0.2 ± 0.1 | 0.27 ± 0.08 |
| **$[Cu_2(Indo)_4(DMF)_2]$ nanocapsules** | 0.17 ± 0.09 | 0.22 ± 0.03 |

Table 2 : Inducible NO synthase activities and nitrite production in intestine from rats treated by RPMI medium (control), empty nanocapsules, indomethacin, $Cu_2(Indo)_4(DMF)_2$ or encapsulated $Cu_2(Indo)_4(DMF)_2$. iNOS activities are expressed in pmol/mg of proteins/hour. Nitrite is expressed as μmol/g of tissue. Results are expressed as mean ± SEM; n = 6 rats per group.

Although gastrointestinal damage was not observed in our experimental protocol, an inflammatory process was demonstrated in these tissues by enhancement of MPO and iNOS activities. $Cu_2(Indo)_4(DMF)_2$ induced a lesser inflammation especially when it was administred after encapsulation. Prostaglandins and NO are recognized as critical mediators of gastrointestinal defence [11, 26]. These mediators modulate mucosal blood flow, mucus release and repair of tissue injury whereas they are able to decrease neutrophil adherence and, thus, neutrophil activation. In our experimental series, copper indomethacinate activated less iNOS than indomethacin, while nitrite production which is the ultimate step of a complex biological cascade, was similarly enhanced. Copper complexes of NSAIDs have already been found more effective than their parent drugs on animal models of inflammation and endowed of antiulcer, anticonvulsivant and anticancer properties [12, 27].

In a previous study, we showed that high doses of copper indomethacinate exerted a slight protective effect on the gastrointestinal tract [16]. This effect seems to be due to free radical scavenging properties more than to a modulation of NOS activity and was strongly potentiated by drug encapsulation. Gastrointestinal inflammation which is the worse side effect of NSAID administration can be decreased by copper complexes and moreover by encapsulation in liposomes or nanocapsules [16]. This might be of interest in sustained treatments of chronic inflammatory diseases.

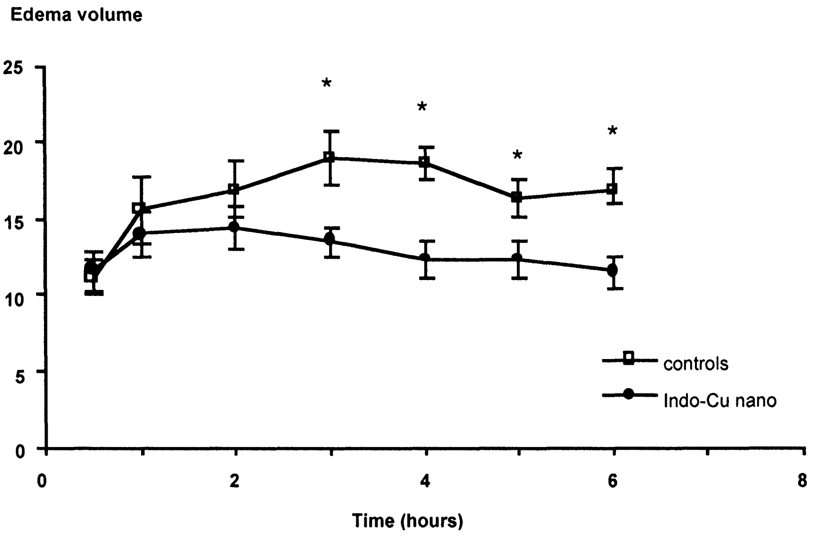

Figure 3 : Time course effect of controls and encapsulated $Cu_2(Indo)_4(DMF)_2$ on carrageenan-induced paw edema. * $p < 0.05$

### 3. Experimental (Materials and Methods)

### 3.1. Synthesis of the copper-indomethacinate complexes.

The complex $Cu_2(indo)_4(DMF)_2$ was synthesized using a modified Sorenson's procedure [12]. Indomethacin (Sigma, Saint-Louis, Mo, USA) (0.716 g, $2x10^{-3}$ mol) was dissolved in 50 ml of

$CH_3OH$ by heating and added to 10 ml of a methanol solution containing $CuCl_2$ (0.134 g, $10^{-3}$ mol). Solution of NaOH ($2.10^{-3}$ mol) was added slowly. After stirring (about 2 hours), the obtained precipitate was collected by filtration, washed with methanol and diethyl ether, dried at 100°C overnight and dissolved in dimethylformamide (DMF). Slow crystallization of this solution gave green plates of compound **I**. However, elongated and green crystals of compound **II** were obtained using the following procedure : indomethacin ($2.10^{-3}$ mol.) and $CuCl_2$ ($10^{-3}$ mol.) were dissolved in DMF (50 ml). NaOH 1M solution ($2.10^{-3}$ mol.) was added slowly. A green solution was obtained and left upon standing. A slow crystallization gave compound **II**.

### 3.2. Preparation of copper-indomethacinate loaded liposomes.

Liposomes were prepared by the method of Stainmesse et al. [28]. The organic phase consisted of lipoïd S75 (200 mg), $Cu_2(Indo)_4(DMF)_2$ (30mg), cholesterol (167 mg), and ethanol (25 ml). This organic phase was poured into an aqueous phase (20 ml) under moderate magnetic stirring. Ethanol was then totally removed under reduced pressure and the suspension concentrated to the convenient final volume under same conditions.

### 3.3. Structure determination

Data for compound **I** were collected on a Stoe IPDS (Imaging Plate Diffraction System) using MoKα radiation (λ= 0.71073 Å). The crystal-to-detector distance was 100 mm. 167 frames (8 min per exposure) were obtained with $0 < \varphi < 250°$ and with the crystals oscillated through 1.5° in φ. Coverage of the unique set was over 95% complete to at least 20.8°.

Table 3. Crystal data and structure refinement

| | $Cu_2(Indo)_4(DMF)_2$ (I) |
|---|---|
| **Crystal parameters** | |
| Empirical formula | $C_{82} H_{74} Cl_4 Cu_2 N_6 O_{18}$ |
| Formula weight | 1700.36 |
| Crystal system | Triclinic |
| Space group | $P\bar{1}$ |
| Unit cell dimensions a, Å | 10.8291(15) |
| b, Å | 13.379(2) |
| c, Å | 16.491(3) |
| α,Å | 105.58(2) |
| β,° | 101.059(19) |
| γ,° | 106.960(17) |
| Volume, $Å^3$ | 2104.6(6) |
| Z | 1 |
| Calculated density ($g.cm^{-3}$) | 1.342 |
| Absorption coefficient ($mm^{-1}$) | 0.701 |
| F(000) | 878 |
| Crystal size | 0.56 x 0.35 x 0.25 mm |
| **Data collection** | |
| Diffractometer | IPDS Stoe |
| Monochromator | graphite |
| Wavelength | 0.71073 Å |
| Temperature | 293(2) K |
| Theta range for data collection | 1.70 to 20.82 ° |
| Completeness to 2theta | 94.8% |
| Ranges of h, k, l | -10 to 10, -13 to 13, -16 to 16 |
| Reflections collected | 11321 |
| Independent reflections | 4193 |
| **Refinement** | |
| Refinement method | Full-matrix least-squares on $F^2$ |
| Data / restraints / parameters | 4193 / 165 / 542 |
| Goodness-of-fit on $F^2$ | 1.029 |
| Final R indices [I>2sigma(I)] | R1 = 0.08, wR2 = 0.22 |
| R indices (all data) | R1 = 0.12, wR2 = 0.24 |
| Largest diff. peak and hole | 0.87 and -0.31 e. $Å^{-3}$ |

Crystal decay was monitored by measuring 200 reflections per image. The final unit cell parameters were obtained by the least-squares refinement of 5000 reflections. Only statistical fluctuations were observed in the intensity monitors over the course of the data collection. Owing to the rather low μR value, 0.24 for **I**, no absorption corrections were considered.

The structures were solved by direct methods (SIR92) [29] and refined by least-squares procedures on $F^2$. For both the compounds, the DMF ligand is severely disordered. It appears on two forms, having the O and N atoms in common. The occupancy factors for the C atoms are in the ratio 0.54(2)/0.46(2)/in **I**. All these disorders were treated using a fully constrained refinement (site occupancies, coordinates, thermal parameters) with the help of the SHELXL-97 program [30]. The H atoms were introduced as riding model. They were given isotropic thermal parameters 20% higher than those of the carbon to which they are attached.

Least-squares refinements were carried out by minimising the function $\Sigma w(F_o^2-F_c^2)^2$, where $F_o$ and $F_c$ are the observed and calculated structure factors. The weighting scheme used in the last refinement cycles was $w=1/[\sigma^2 (Fo^2)+(XP)^2]$ where $P=(Fo^2+2Fc^2)/3$ and X= 0.1448 for **I**. Details of data collection and refinement are given in Table 3.

Calculations were performed with the SHELXL-97 programs [30] running on a PC. The drawing of the molecule was obtained using CAMERON [31]. The fractional atomic coordinates and the equivalent thermal parameters for all atoms but H, anisotropic thermal parameters for non hydrogen atoms and atomic coordinates for H atoms have been deposited at the Cambridge Crystallographic Data Centre.

### 3.4 Biological assays

### 3.4.1.Animals and treatments

Animals Male Sprague-Dawley rats (Dépré, Bourges, France) of 160 to 180 g mass were fed standard laboratory chow (UAR Villemoisson/Orge, France) and water *ad libitum*.

Treatment-protocol.

Rats were daily administered by gavage at the same hour and decapitated on the 8th day . Peritoneum was opened and 30 cm of intestine above the ileo-Cæcal valve were collected. This tissue was rinsed with phosphate-buffer saline (PBS pH = 7.4), opened along the antimesenteric border and observed to quantify possible ulcerations. One cm-length pieces were taken 10, 20 and 30 cm above the ileo-cæcal valve, and pooled for tissular determination of myeloperoxidase (MPO) and nitric oxide synthases (NOS) activities. Tissue culture *ex-vivo* for five hours led to collection of supernatants in which nitrite amount was determined.

3.4.2. Intestinal damage scores.

Jejuno-ileum ulceration was round, segmental and sometimes confluent. Damage was scored by tracing the outline of ulcerated areas from two-fold magnified photographic images onto paper, and massing the cut-outs. Jejuno-ileum damage score (expressed in %) was expressed as the ratio: sum of ulcerated areas/total photographed area x 100 [32].

3.4.3. NOS activities in tissue samples.

Tissue iNOS activity was estimated by measuring the conversion of L-$^{14}$C-arginine to L-$^{14}$C-citrulline as described by Bush et al. [33]. Tissue samples (approximately 250 mg) were homogenized in buffer (pH 7.4) containing 50 mM Tris-HCl, 1 mM dithiotreitol, 23.4 μM leupeptin, 14.6 μM pepstatin and 1 mM phenylmethylsulfonylfluoride (PMSF). After sonication on ice and centrifugation at 250 g for 30 min at 4°C, 100 μl of the supernatant were added to a reaction mixture containing 50 mM TrisHCl (pH 7.4), 1.58 μM L-$^{14}$C-arginine, 200 μM NADP, 10 μM flavin mononucleotide, 10 μM flavine adenine dinucleotide, 1 mM dithiotreitol, 50 μM tetrahydrobiopterine and 50 mM valine, with 2 mM $CaCl_2$ (total NOS activity) or 1 mM EDTA and 1 mM EGTA (iNOS activity). After 10 min of incubation at 37°C, the enzymatic reaction was terminated by adding cold phosphate buffer (pH 5.5) containing 3 mM EDTA. L-$^{14}$C-citrulline was separated by applying samples to columns containing pre-equilibrated Dowex AG50W-X8, eluting them with water and measuring the amount of radioactivity by means of scintillation counting. Protein content was measured according to Lowry [34]. Enzyme activity is expressed as picomols of citrulline formed per milligram of protein per hour.

### 3.4.4 Culture ex-vivo.

After the sacrifice of rats, intestinal tissue was carefully washed three times in sterile and apyrogen normal saline, then cultured in a 24-well plate (Nuncleon, France) with 1.6 ml of RPMI 1640 medium (Sigma, Saint-Louis, Mo, USA) without phenol red and supplemented with penicillin

(100 IU/ml)/streptomycin (100 μg/ml). Tissues were cultured for 5 hours in a humid atmosphere at 37¡C with 5% $CO_2$. After that time, supernatants were collected and kept at -80°C before determination of nitrite amounts.

**3.4.5. Nitrite measurement in tissue culture supernatants.**

Nitrite was measured as an indicator of NO formation [35]: concentration was determined in duplicate spectrophotometrically using the Griess reagent (1% sulfanimide, 0.1% naphthylenediamine dihydrochloride, and 2.5% $H_3PO_4$) [36]. Final concentrations were expressed as mmole/mg of tissue.

**3.4.6. MPO activity in tissue samples.**

MPO activity was measured as described by Maehly and Chance [37]. MPO was extracted from tissue by suspending the material in lysis buffer containing 20 mM $KH_2PO_4$ and 1.4 mM hexadecyltrimethyl ammonium bromide (HTAB) (pH 6.0), before homogenization on ice with a Polytron homogenizer. Homogenates were freezed and thawed twice, sonicated on ice 30 sec and centrifuged. The supernatant was assayed spectrophotometrically for MPO activity: 500 μl of suspension was combined with 2500 μl of buffer (pH 6.0) containing 0.16 mM $Na_2HPO_4$, 18.4 mM $KH_2PO_4$, 44.8 μM guaicol and 0.04% hydrogen peroxide. The kinetics of absorbance at 470 nm was recorded with a spectrophotometer thermostated at 40°C. One unit of MPO activity is defined as that degrading 1 μmole of peroxide per minute. Change in absorbance for each sample was expressed in international units (IU) by using a standard curve of horseradish peroxidase established in the same experimental conditions. MPO activity is expressed as milliunits (mU) per milligram of total protein, measured according to Lowry [34].

**3.4.7. Carrageenan-induced paw-edema.**

Rats were fasted overnight and were treated orally with indomethacin or copper-indomethacinate-liposomes (15 mg/kg) or the vehicle. Rats were refed immediately after the treatment. One hour later, a 0.1% solution of lambda carrageenan (kindly provided by Dr Willougby, London, UK) (0.1 ml) was injected into the right hind foot pad. The volume of the edema was recorded by means of plethysmography after carrageenan injection and every hour thereafter for 5 hours. Changes in paw volume relative to the measurement taken just after carrageenan administration were calculated.

**3.4.8.Statistical analysis.**

The results were expressed as mean ± SEM. The results were analyzed with ANOVA procedure. If the ANOVA procedure yielded a significant interaction between variables, comparisons between two groups were made with the Mann-Whitney U test. A statistical difference between groups was considered significant when $p \leq 0.05$.

**Acknowledgements**

The authors wish to thank M. Lenoir and H. Gornitzka for their excellent technical assistance.

**References**

[1] R. L .Ernst, J. W. Hay,. *Am. J. Public Health*, **84** (1994) 227.
[2] R. F. Clark, A. M. Goate, *Arch. Neurol.*, **50** (1993) 1164.
[3] P. L. McGeer, E. G. McGeer, J. Rogers, J. Sibley, *Lancet*, **335** (1990) 1037.
[4] J. C. S. Breitner, K. A. Welsh, M. J. Helms, P. C. Gaskell, B. A. Gau, A. D. Roses, M. A. Pericak-Vance, A. M. Saunders, *Neurobiol. aging*, **16** (1995) 523.
[5]A. Klegeris, P. L. McGeer, *J. Neuroimmunol.*, **53** (1994) 83.
[6] J. Rogers, L. C. Kirby, S. R. Hempelman, *Neurology*, **43** (1993) 1609.
[7] R. Udassin, I. Ariel,Y. Haskel, N. Kitrassky, M. Chevion, *Free Radic. Biol. Med.*, **10** (1991) 1.
[8] D. J. Schmeling, R. A. Drongowski, A. G. Coran, *Prog. Clin.. Biol. Res.*, **299** (1989) 53.
[9] J. Schreiber, G. L. Foureman, M. F. Hughes, R. P. Mason, T. E. Eling, *J. Biol. Chem.*, **264** (1989) 7936.
[10] J. R. J. Sorenson, *Prog.Med.Chem.*, **26** (1989) 437
[11] C. Scarpignato, in "*Recent Advances in Pathophysiology of Gastrointestinal and Liver Diseases*", (J.-P. Galmiche, J. Gournay Eds) J. Libbey Eurotext, Paris, **47** (1997) 84.
[12] J. R. J. Sorenson, *J. Med. Chem.*, **19** (1976) 135.
[13] J.R.J. Sorenson U.S. Patent # 5,082,834 (1992}.
[14] V.Brumas, B.Brumas, G.Berthon, *J. Inorg.Biochem.***57** (1995) 191.
[15] U.Weser, K-H.Sellinger, E.Lengfelder, W.Werner, J.Strahle, *Biochem. Biophys. Acta* **631** (1980) 232.

[16] V. Bertrand, F. Guessous, A. L. Le Roy, B. Viossat, H. Fessi, A. El Abbouyi, J.-P. Giroud, M. Roch-Arveiller (private communication).
[17] S. Moncada, A. Higgs, *New England J. Med.*, (1993) 2002.
[18] D. Salvemini, T. P. Misko, J.-L. Masferrer, K. Seibert, M. G. Currie, P. Needleman, *Proc. Natl. Acad. Sci. USA.*, **90** (1993) 7240.
[19] I. Alican, P. Kubes, *Am. J. Physiol.*, **270** (1996) G225.
[20] J. D. Lee, A new concise Inorganic Chemistry 3rd ed. (1986) 379. London van Nostrand Reinhold.
[21] T. Kawata, H. Vekusa, S. Ohba, T. Furukawa, T. Tokii, Y. Muto, M. Kato, *Acta Cryst.*, **B48** (1992) 253.
[22] D. Kovala-Demertzi, A. Theodorou, M. A. Demertzis, C. P. Raptopoulou, A. Terzis, *J. Inorg. Biochem.*, **65** (1997) 151.
[23] C. Dendrinou-Samara, D. P. Kessissoglou, G. E. Mannoussakis, D. Mentzafos, A. Terzis, *J. Chem. Soc. Dalton Trans.* (1990) 959.
[24] T. J. Kistenmacher, R. E. Marsh, *J. Am. Chem. Soc.*, **94** (1972) 1340.
[25] K. Frenkel, F. Blum, W. Troll, *J. Cell. Biochem.*, **30** (1986) 181
[26] S. K. Konturek, P. C.Konturek, *Digestion,* **51** (1995) 1.
[27] J. R. J. Sorenson in "*Ulcer diseases*" S. Szabo and C. J. Pfeiffer eds. CRC Press. Boca Raton (Fl) (1989) 357.
[28] S. Stainmesse, H. Fessi, J.-P. Devissaguet, F. Puisieux, European patent (1989) n° 894018571.
[29] A. Altomare, G. Cascarano, G. Giacovazzo, A. Guagliardi, M. C. Burla, G. Polidori and M. Camalli, SIR92 - a program for automatic solution of crystal structures by direct methods. *J. Appl. Cryst.* **7** (1994) 435.
[30] G. M. Sheldrick, SHELX-97, program for crystal structure refinement, University of Gottingen, 1997.
[31] D. J. Watkin, C. K. Prout, L. J. Pearce, *CAMERON*, Chemical Crystallography Laboratory, University of Oxford, Oxford, (1996).
[32 ] J. L. Wallace, W. Mc Knight, M. Miyasaka, T. Tamatani, J. Paulson, D. C. Anderson, D. N. Granger, P. Kubes, *Am. J. Physiol.*, **265** (1993) G993.
[33] P. A. Bush, N. E. Gonzalez, J. M. Griscavage, L. J. Ignarro, *Biochem. Biophys. Res. Commun.* **185** (1992) 960.
[34] O.H. Lowry, *J.Biol.Chem.*, **193** (1951) 265.
[35] M. A. Marletta, P. S. Yoon, R. Ivengar, C. D. Leaf, J. S. Wishnok, *Biochemistry,* **27** (1988) 8706.
[36] N. J. Freehold, in *Worthington Biochemical Corp.* (1972) 43.
[37] A. C. Maehly and B. Chance, The assay of catalases and peroxidases. In "Methods of Biochemical Anazlysis" S. Glick ed, Interscience New York, (1954) 357-408.

**Received: October 19, 1998 - Accepted: November 16, 1998 - Received in revised camera-ready format: November 19, 1998**

